# On the Structure and Growth of Dentine

**Published:** 1877-08

**Authors:** Chr. Sihler


### THE DENTAL REGISTER.

VOL. XXXI.] AUGUST, 1877. [No. 8.

### ON THE STRUCTURE AND GROWTH OF DENTINE.

BY CHR. SIHLER, M. D.

"There are few anatomical questions which have called forth a more lively controversy than the structure and growth of dentine."—Beale. The structure of the simple tissues.—Lectures before the Royal College of Surgeons.

Although the writer of this little essay is not a dentist, but an ordinary practitioner, yet he thinks that the structure and growth of teeth is one of the most interesting of subjects in the domain of histological science. They are of so much interest because such a variety of different tissues enter into their formation, tissues of different histological character, of different methods of of growth and of different physiological functions. The teeth are further attractive on account of the beauty and regularity of their structure. But the points which the writer wishes preeminently to mention, and the reasons why he feels himself under special obligations to these structures are, that teeth in their development show so clearly, and unmistakably, certain general principles in the structure, the growth and the removal of other tissues. So that these organs may not only be instrumental in cracking the nuts of ordinary life, but also in cracking some of the nuts of science.

For while dentine is the most perfect and distinct representative of the connective tissue class—providing this shall be upheld yet as such—the formation of the dentine and cementum furnishes the key for the solution of the much vexed question of the formation of bone, and especially of that of the construction of the canaliculi, while the removal of the deciduous teeth demonstrates unmistakably the *true* physiological method employed in the removal of other tissues, (the principle of the substitution of tissues—Virchow's Cellular Pathology) the formation of the medullary cavity in bone and preeminently makes clear the process of intracartilagenous ossifications.

But though the interest which thus seems concentrated in the teeth offers ample reasons for the investigation of these structures, it does not furnish an excuse for writing on this subject. Such an excuse, however, we find in the quotation from Beale, which is placed at the head of this paper, and in the difference of views which we find in the works treating on our topic.

The teeth and the process of teething offer of course a series of interesting problems for investigation. The human tooth for example, is made up of three different kinds of hard tissues; there is further the pulp-cavity with its contents, vessels, nerves and embryonic or young "connective" tissue; upon this hitches the question of what nature is the connection between the pulp and dentine, and how do the nerves end in the pulp? Then where does the enamel come from and how does its development differ from that of dentine and cementum? Are the extraordinary statements in regard to the structure and history of the enamel organ true? How are the deciduous teeth removed; how are the jaws changed in size and configuration? We see there are quite a number of interesting questions. In this paper, however, we shall confine ourselves to the dentine and cementum.

Now although these tissues, the dentine and cementum, offer no special difficulties for investigation, a variety of opinions have been and are held in regard to their structure and growth. As the cause for these differences cannot be found in the obstacles for examination, they must be sought in the method of reasoning which has been brought to bear npon the facts. The writer endeavors, in this paper, to prove every assertion he makes, and takes the liberty to point out, when presenting the views of others, why these seem not true; but in following this method he will be obliged often to repeat and to appear at times unnecessarily plain, for which faults he begs the reader's indulgence here in the beginning.

The histologist has too objects in his investigations. He may work in the spirit of the descriptive anatomist, or he may work in the spirit of the physiologist proper. When working in the former, he continues or follows out with his needles, razor, section cutter, staining and chemicals, the work which the anatomist began with his scalpel and scissors; he wishes to gain an insight into the structure of the tissues, whether a tissue is homogeneous, fibrous, or granular; whether the fibres run in one direction or another; whether any given substance is simple or compound; how the capillaries are arranged in the different organs; how the nerves end, the lymphatics begin, and so on. Of course the results of such investigations are of as much interest to the physiologist as the knowledge of structure of parts visible to the naked eye. But, as remarked, the histologist may have another aim, he may wish to study the processes that occur in the body, the changes, growth, development, decay; he wishes to follow the organism from its beginning, watch the different methods of growth, where, when and whence the different organs and structures originate; what their life history is, what the conditions under which the different forces work together to bring about the final result, and last but not least, what the most simple and ultimate actions, processes and powers are, which, when working together bring about the final result.

Of course the processes themselves can never, or hardly ever, be observed; hence they can only be ascertained by inference from the structure, and by making observation upon tissues while growing and developing, and by noting the changes.

The present paper on the structure and growth of dentine has thus been divided into three sections. The first describes dead and dry dentine, the second treats of living and growing dentine, the third briefly offers some opinions on the growth and development of this tissue.

### I. DEAD AND DRY DENTINE.

It is very important in thinking on and speaking of hard tissues, to keep in the mind of one's self and before the eyes of those you are addressing, whether the dead or the living tissue is the subject of your thought and demonstration. This is not always done, and one is often in doubt which of the two is meant; and at times one finds that writers speak of properties and peculiarities which are attributable to one of the conditions only. In this section we will speak of dead and dry dentine only. To study its main features it is well to make a longitudinal section through an incisor or cuspidate tooth. It may be made with a jeweler's saw, and if one wishes to preserve also the enamel perfectly, it is a good plan—after having filed, ground and polished down one side, to imbed the section, with the aid of heat, in some Canada Balsam on a little square of glass, and then to grind down the other side, the edges of the enamel can thus be protected, and as the section is fixed on glass, repeated examinations can readily be made whether the desired thinness has been attained. Such a section will show the general construction of a tooth, the different tissues entering its formation, enamel, cementum and dentine, and their mode of union. If such a section be examined dry, under the microscope, it will be found that the dentine is not a solid mass, but that dark lines running from the pulp cavity outward, show that it is perforated by numerous canals. That the dark lines mean canals containing air and not solid fibres which would not let the light pass through them, can be proved by adding a little turpentine to such a section, or some colored fluid. Then it can be seen that the air is expelled and that the fluids take its place.

Some may consider it pedantic to prove that these black lines signify canals and not solid rods. But the history of the views on the structure of bone will justify and excuse me. When the lacunæ and canaliculi were first discovered, the former were named bone corpuscles, because they had a dark appearance and on that account were considered to be solid bodies, likewise the canaliculi were called calciphori, because they were considered the depositories of the lime-salts.

The direction of the canals is in the main a straight one, often near their pulp extremity they are bent a little downward, and near their outer extremities a little upward, they present also frequently some faint undulations, so that their course would be slightly spiral. The calibre of the canals is not uniform, they are wider nearer the pulp cavity, and decrease in width as they approach the cementum or enamel. They are further provided with side branches, which are more abundant and finer outwards, larger and less plentiful the nearer we come to the pulp cavity. Beneath the cementum and enamel there is frequently a stratum where the structure is regular, the canals end there in small irregular cavities, which territory might be signified irregular dentine. The appearance which this territory presents has also been termed "granular."

Of an extension of the dentinal tubules into the enamel I have not yet seen anything; appearances which justify the view that there is a structural unity must be rare nor would the history ot development speak for such views. From the latter we might expect, and do find in fact, the same relation between the dentine and enamel, which exists between the true skin (the derma) and the epidermis; the enamel is modified epidermis, and the dentine modified "white fibrous tissue," the tooth, as a whole, being of course a modified dermal papilla. Although a distinct line can mostly be recognized, forming the boundary line between the dentine and enamel, and although one may say there is a superposition, yet for all that, the union may be as intimate as though they formed an undistinguishable unit, for we must keep in mind that the ready made enamel was not laid over the ready made dentine, as plastering is laid upon lath, but that there never was any third something between the two, that they have been grown together. Their union is more like that of two pieces of iron welded together.

The cementum which covers the roots of the teeth is a lime conducting tissue which is characterized by oval roundish irregular excavations (lacunæ) which it contains, which are in communication with each other through numerous canals. Cementum generally is considered to be bone, there is however, frequently, this difference to be. observed between it and the true bone as we find it for example in the long bones that the canaliculi are less regular in their arrangement and at times so wide that they remind one of the dentinal canals, nor is the arrangement and form of the lacunæ as typical and methodical as we find it in bone. Cementum as we find it, especially in older teeth, is not rarely a tissue, half physiological, half pathological, and bears some resemblance in this respect to the callus formed on the hands of a laboring man. The more hardships a tooth had to go through and the oftener its root is attacked by inflammation, the thicker will the cementum be. Such cementum shows distinct strata, indicating that its growth has been a periodical one. If I had to give a short answer to the question, what is cementum? I would say it is the ossified tooth-sac.

Now while a distinct line can generally be recognized between the enamel and dentine, there is nothing analogous between the cementum and dentine. These two tissues, as a rule, pass one into the other without letting the observer know exactly where one begins and the other ends; often there is found between the territory which is plainly dentine and that which is plainly cementum, a stratum which is almost homogeneous and of which it is difficult to say what it may be. The history of development makes this condition clear enough too, because both tissues are produced by the so called middle layer of the embryo and because the tissue producing the first strata of cementum and dentine form a continuous mass, a fact of which every one can convince himself if he will examine the lower extremity (root) of a growing tooth, the pulp and tooth-sac are there in continuity,—form one tissue. So much about the dentinal canals and the mode of connection between the dentine and enamel and the dentine and cementum. As far as the general mass of the dentine is concerned that is perfectly homogeneous and shows no more of structure than it does to the naked eye. The lime salts which give the hardness to the dentine are so ultimately united with the organic matrix, that this shows nothing of granulation, as the calcified cartilage for example presents them.

The mere inspection of dead and dry dentine, without having used chemical reagents has shown us that dentine appears to be a homogeneous substance perforated by numerous, canals, which by their smaller side branches stand in connection with each other.

The question now arises, are there special walls to these canals, or are they merely channels in a perfectly homogeneous mass? If there are such special walls then we have the right to speak of dentinal *tubules*, if not, then we can speak only of *canals*. For there is certainly a difference expressed by these two terms. If one bores holes into wood he has a canal but by no means a tube; if he fits a gas-pipe into such an excavation then he can speak of a tube. So far the examinations of the dentine have not justified us in believing in the presence of special walls to the canals, but if we treat a longitudinal section, as we examined just now, with strong muriatic acid we find that dentine swells up, becomes pultaceous, soft, and the general mass of it disappears while there remain elements corresponding to the position and course of the dentinal canals. These elements appear as white glistening fibres, and by pressing upon the covering glass, stretching them, etc., one is forced to the conclusion that they are very tough, dense and elastic. They will also resist the action of the strong muriatic acid for quite a while, finally however, they are destroyed by this agent. One might say upon seeing them, these are now the dentinal tubules. But before making this statement let us ask first are these really tubules? are they not solid fibres which, having been dried down have rested unobserved in the dentinal canals and were now isolated by the acid? To demonstrate that these elements are really tubular one can procure such a section as will show the opening of the dentinal canals in the walls of the pulp cavity, or he can make selections running across the canals, and treat such with strong acid, then he can *see* that these elements which he isolates are distinctly tubular. After such investigations, and then only, we are justified in speaking of dentinal tubules.

Some of the authors seem to consider it self-evident that tubules are present in dentine. Tomes, for example, begins his description of dentine thus: "If a median longitudinal section of a tooth be made, it will be seen that the surface of the central cavity is every where pierced by an infinite number of extremely minute openings. They are the orifices of the dentinal tubes, parietes of which, together with the matris in which they lie, make up the walls of the pulp-cavity," etc.

Now if Tomes had thought it worth while to demonstrate the tubular, instead of assuming them, he would not have thought of offering certain evidence for the presence of a soft fibri within the tubule. For some of the phenomena which he produces as proofs of the presence of the soft fibril are the same that we have adduced to demonstrate the presence of the tubule. But this point will be discussed further on more fully.

The next question is what is the nature of the material of which the dentinal tubule is made, and what the nature of the intertubular matrix? If a section of cementum or bone is treated with strong muriatic acid, it behaves similar to dentine, the general mass of these tissues is dissolved while the walls of the lacunæ and canaliculi resist the action of the acid as the dentinal tubules do. Further, if a section of tendon or some of the fibrous tissue of the peritoneum is thus treated, a homogenous mass which swells up a good deal at first is likewise dissolved and glistening fibres are isolated. The examination of these tissues show that a homogenous substance and a dense elastic material in the form of fibres, or tubes, enter into their formation; and the peritoneal tissue may lead us to the tissue where we find one of these elements in a pure state, namely to the ligamentum nucha, the yellow and dense band in the neck of rumenants are especially well developed on account of the weight it has to support. This consists of nothing but fibres—the yellow elastic fibre of the books, a texture of which also the large blood-vessels, avona, etc., are mainly constructed—which are of extreme denseness, firmness, somewhat elastic, and have an almost incredible resistance against powerful chemicals, acids and alkalies. For over twenty-four hours the fibres of this ligament can be kept in strong muriatic acid without being destroyed. As the dentinal tubules show the same chemical and physical properties as the yellow elastic fibres, as they further apparently have to bestow denseness and elasticity to the tooth we are justified in classifying the tubule with the yellow elastic fibre. The color of worn down dentine also has a striking resemblance to the yellow elastic tissue when we see it in mass, and as the tubules are in such dentine perfectly solidified, this would explain the yellow color. This is, however, merely a suggestion. As far as the intertubular matrix is concerned this may also be obtained by boiling the decalcified tooth, and is found to be a substance of the nature of animal glue.

As regards the question, whether the tubules are calcified. It is difficult to give a positive answer. To judge from the nature of the tubule, its extreme denseness would make it seem almost impossible to receive any limesalts, nor is there any evidence that the elastic fibre ever does. Two arguments have been brought forth to support the idea that they are calcified. *First.* The tubules have been shown to exist in teeth about two thousand years of age, and it has been argued that all the soft portions must have been decomposed. Second. Teeth have been exposed to the process of putrefaction and treated with strong alkalies, and these tubules have, after that, been isolated, and it has been said that all the soft portions of the tooth must have been destroyed. Why these proofs do not seem sufficient to the writer, he will try to show further on.

The investigation of the dead and dry dentine then, to sum up, has shown, that this tissue is made up of two kinds of materials, a homogenous substance of the nature of glue in which the limesalts are deposited, and a substance of the nature of the yel-elastric fibre which lines the canals and canaliculi, forming the dentinal tubule.

We have learned further that bone and cementum are constructed similarly and that these same elements, or similar and analogous ones, enter into the formation of other tissues.

We have learned also that there is some difficulty in distinguishing fibrils and tubules and that it is always better to demonstrate any thing than to assume that it is present.

